# Accumulation of Biomass and Mineral Elements with Calendar Time by Cotton: Application of the Expanded Growth Model

**DOI:** 10.1371/journal.pone.0072810

**Published:** 2013-09-09

**Authors:** Allen R. Overman, Richard V. Scholtz

**Affiliations:** Agricultural and Biological Engineering Department, University of Florida, Gainesville, Florida, United States of America; BASF Cropdesign, Belgium

## Abstract

Accumulation of plant biomass (Mg ha^−1^) with calendar time (wk) occurs as a result of photosynthesis for green land-based plants. A corresponding accumulation of mineral elements (kg ha^−1^) such as nitrogen, phosphorus, and potassium occurs from the soil through plant roots. Field data from literature for the warm-season annual cotton (*Gossypium hirsutum* L.) are used in this analysis. The expanded growth model is used to describe accumulation of biomass and mineral elements with calendar time. The growth model predicts a simple linear relationship between biomass yield and the growth quantifier, which is confirmed with the data. The growth quantifier incorporates the unit processes of distribution of solar energy which drives biomass accumulation by photosynthesis, partitioning of biomass between light-gathering and structural components of the plants, and an aging function. A hyperbolic relationship between plant nutrient uptake and biomass yield is assumed, and is confirmed for the mineral elements nitrogen, phosphorus, and potassium. It is concluded that the rate limiting process in the system is biomass accumulation by photosynthesis and that nutrient accumulation occurs in virtual equilibrium with biomass accumulation. The expanded growth model describes field data from California and Alabama rather well. Furthermore, all model parameters were common for the two sites with the exception of the yield factor *A* which accounts for differences in soil types, environmental conditions, fertilizer levels, and plant population.

## Introduction

The authors have published a growth model describing accumulation of plant biomass and mineral elements with calendar time for agricultural crops [1]. The model was first developed to describe crop biomass with time in response to capture of solar energy by photosynthesis. It was later expanded to include mineral elements (such as nitrogen, phosphorus, and potassium). First application was to warm-season perennial grasses such as bermudagrass (*Cynodon dactylon* L.) and bahiagrass (*Paspalum notatum* Flügge). It was later shown to apply to the annual corn (*Zea mays* L.).

More recently we published a simplified theory of biomass production by photosynthesis [2]. The theory is structured on a rigorous mathematical framework and a sound empirical foundation using data from the literature. Particular focus is on the northern hemisphere where most field research has been conducted, and on the warm-season perennial coastal bermudagrass for which an extensive database exists. Three primary factors have been identified in the model: (1) an energy driving function, (2) a partition function between light-gathering (leaf) and structural (stem) plant components, and (3) an aging function. These functions are then combined to form a linear differential equation. Integration leads to an analytical solution. A linear relationship is established between biomass production and a growth quantifier for a fixed harvest interval. The theory is further used to describe forage quality (nitrogen concentration and digestible fraction) between leaves and stems of the plants. The theory can be applied to annuals (such as corn) as well as perennials. Crop response to various applied elements (such as nitrogen, phosphorus, potassium, calcium, and magnesium) can be described. The theory contains five parameters: two for the Gaussian energy function, two for the linear partition function, and one for the exponential aging function.

The work has been expanded to describe response of corn to plant population and absorption of solar energy within the plant canopy [3]. A simple exponential model coupling biomass yield with plant population was used to analyze data from three field studies. One of the studies reported data on absorption of solar energy within the plant canopy, which provided a rational basis for the simple exponential model. The growth model has also been used to describe high quality data from a field study at Florence, SC, USA [4]. The planting time of April 2 maximized capture of solar energy for biomass production. A hyperbolic relationship between plant nutrient uptake and biomass yield was assumed and confirmed by the analysis for the mineral elements nitrogen, phosphorus, and potassium. It was concluded that the rate limiting process in the system was accumulation of biomass by photosynthesis and that nutrient accumulation occurred in virtual equilibrium with biomass accumulation. This study will focus on the application of the expanded growth model to warm-season annual cotton. This work serves as an example of the model' robust nature and ability to describe the growth of a dicotyledonous species, going beyond its ability to describe the growth forage monocotyledons from which the model was originally developed.

## Methods

The first step is to define relevant quantities (variables and model parameters): *t* is calendar time (referenced to Jan. 1), wk; *Y* is biomass yield (dry matter), Mg ha^−1^; *N_u_* is plant nutrient uptake (N, P, or K), kg ha^−1^; *N_c_* = *N_u_*/*Y* is plant nutrient concentration (N, P, or K), g kg^−1^. A common reference time is used to facilitate comparison among various studies. The second step is to utilize a mathematical model which relates biomass accumulation to calendar time. For this purpose we adopt the expanded growth model discussed by Overman and Scholtz Section 3.5 of [1]), which can be written as
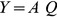
(1)with *A* as a yield factor, Mg ha^−1^ and *Q* as a dimensionless *growth quantifier*. The growth quantifier is defined as
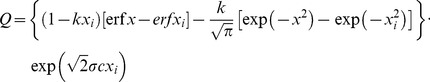
(2)with x, the dimensionless time variable, defined by
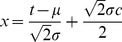
(3)with the parameters in Eq. (2) and Eq. (3) include: 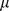 is the time to the peak of the solar energy distribution, wk; 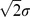 is the time spread of the distribution, wk; k is the partition coefficient between light-gathering and structural components of the plants, and c is the aging coefficient for the plant species, wk^−1^. The reference state of the system, xi, corresponds to the time of initiation of significant plant growth ti, wk. The ‘error function’ or erf x, in Eq. (2) is defined by
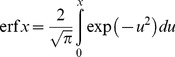
(4)where u is the variable of integration for a given Gaussian distribution 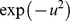. Values of the erf x can be found in Table 7.1 of Abramowitz and Stegun [5] or from other like handbooks of mathematical functions.

Examination of data of coupling between plant nutrient accumulation *N_u_* and plant biomass Y leads to the hyperbolic phase relation
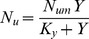
(5)with *N_um_* as potential maximum plant nutrient accumulation at high *Y* and *K_y_* is the value of *Y* at which *N_u_ = N_um_*/2. This subject is explored in more detail in the next section of application to field studies in California and Alabama.

## Results

### California Study

Data for this analysis are taken from a study in the San Joaquin Valley, CA by Fritschi et al. [6] with ‘Acala’ cotton grown on Wasco sandy loam (coarse-loamy, mixed, nonacid, thermic Typic Torriorthent) in 1998, 1999, and 2000. The last year of the study is chosen where applied nitrogen was 0, 56, 112, 168, and 224 kg ha^−1^. The authors state that weather conditions were considered good for cotton growth. Total irrigation of approximately 1.0 m was supplied in six applications. All treatments were replicated four times. Planting date was approximately April 1 (*t* = 12.9 wk), with plant samples collected at *t = *21.9, 26.4, 30.1, and 35.1 wk.

**Figure 1:**
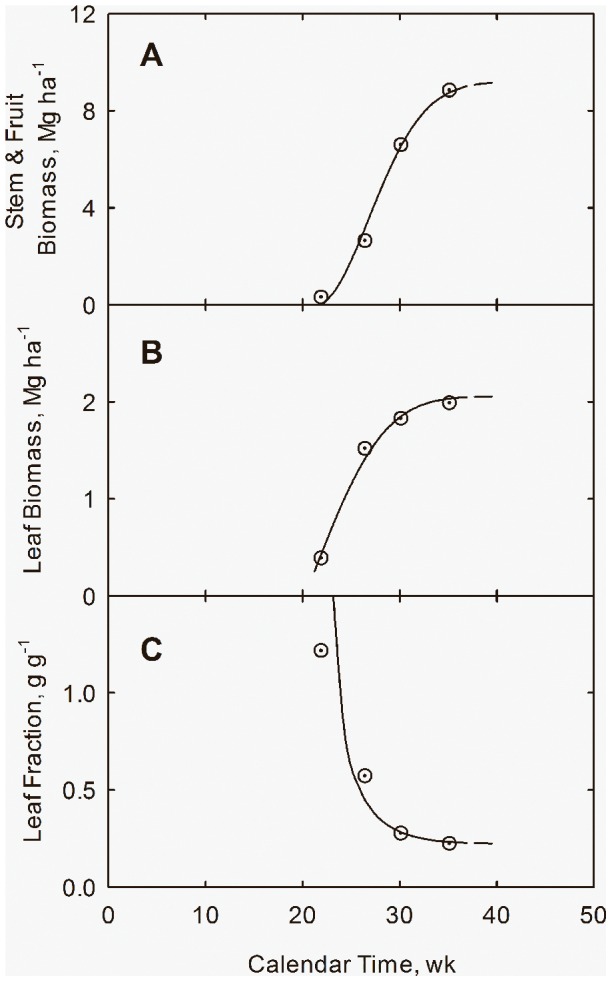
Biomass Accumulation with calendar time (*t*) at applied N = 168 kg ha^−1^ for cotton in San Joaquin Valley, CA. Accumulation of stem + fruit (*Y_S_+Y_F_*) (**A**) and leaf (*Y_L_*) biomass (**B**), and leaf fraction (*f_L_*) (**C**) with calendar time. Data adapted from Fritschi et al. (2003). Curves drawn from Eqs. (7) through (10).

Results for N** = **168 kg ha^−1^ are listed in Table 1 and shown in Figure 1. Biomass was partitioned into three components: leaves (*Y_L_*), stems (*Y_S_*), and fruit (*Y_F_*). Leaf fraction is defined as *f_L_* = *Y_L_*/(*Y_L_+Y_S_+Y_F_*). Time is referenced to Jan. 1. From Figure 1 time of significant growth is estimated to be *t_i_* = 21.2 wk. It is also assumed that *x_i_* = 0, which maximizes the capture of solar energy by the plants. Following Overman and Scholtz [3] model parameters are assumed to be: 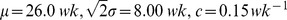. The dimensionless time variable is now given by 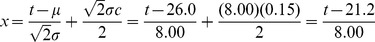
(6)


**Table 1:** Accumulation of leaf (*Y_L_*), stem (*Y_S_*), and fruit (*Y_F_*) biomass and leaf fraction (*f_L_*) with calendar time (*t*) by cotton on Wasco sandy loam at *N* = 168 kg ha^−1^ in San Joaquin Valley, CA (2000) [6].

*t*	*x*	erf *x*	exp (−*x* ^2^)	*Q*	*Y*	*N_u_*	*P_u_*	*K_u_*	*f_L_*
wk					Mg ha^−1^	kg ha^−1^	kg ha^−1^	kg ha^−1^	
21.2	0.000	0.000	1.0000	0.000	0.00	-	-	-	-
21.9	0.088	0.099	0.9924	0.039	0.14	0.39	0.32	-	-
26.4	0.650	0.642	0.655	1.75	2.39	1.52	1.74	0.91	0.549
30.1	1.112	0.884	0.290	3.61	4.49	1.83	2.35	4.25	0.217
35.1	1.738	0.986	0.0488	4.83	5.82	1.99	2.48	6.37	0.184
	∞	1	0	5.08	6.08	-	-	-	-

The growth quantifier for the light-gathering component of the plant (*Q_L_*) is given by 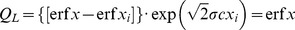
(7)


**Figure 2:**
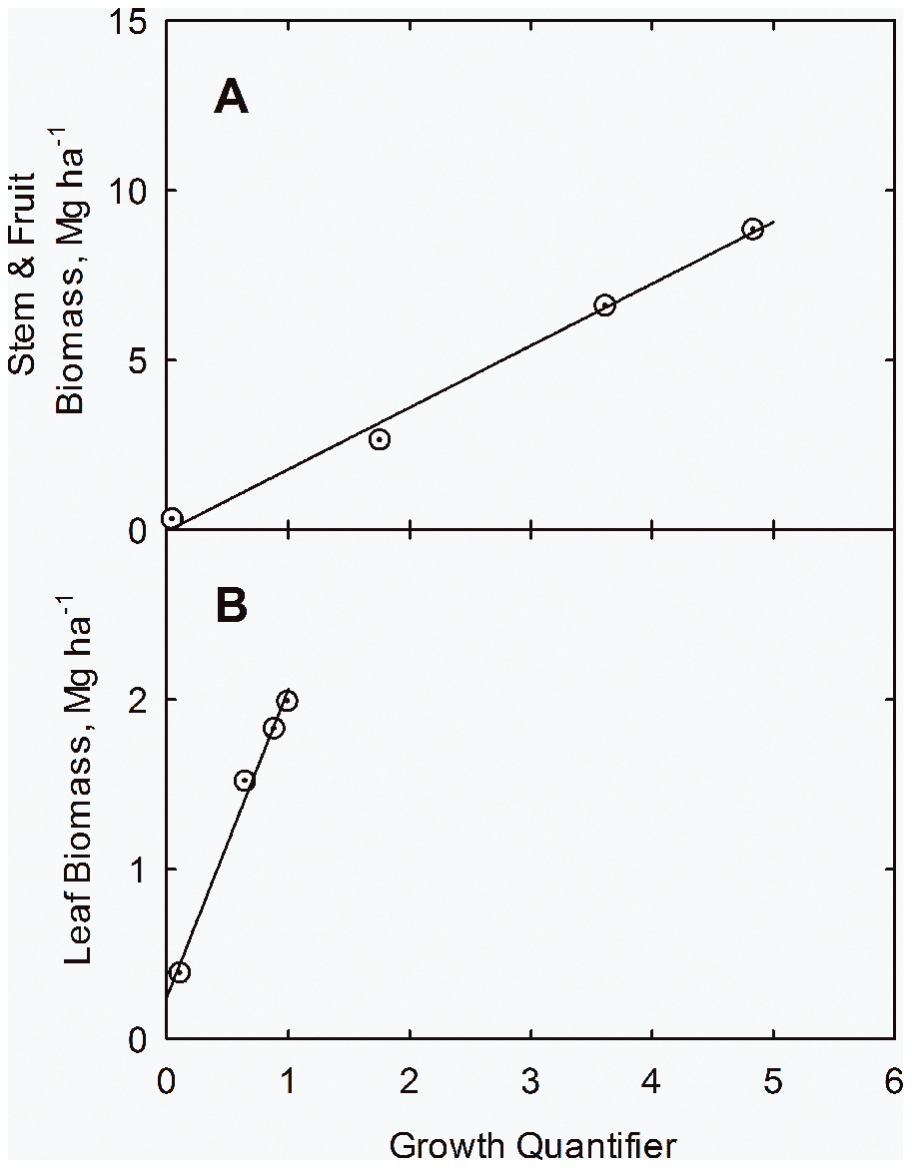
Biomass Accumulation linearization with the growth quantifier (*Q*) at applied N = 168 kg ha^−1^ for cotton in San Joaquin Valley, CA. Correlation of stem + fruit (*Y_S_+Y_F_*) (**A**) and leaf (*Y_L_*) biomass (**B**) with the growth quantifier. Yield data adapted from Fritschi et al. (2003). Lines drawn from Eqs. (8) and (10).

Leaf biomass can now be linked to *Q_L_* by linear regression of values in Table 1

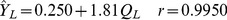
(8)with a correlation coefficient of *r* = 0.9950. Note that the intercept in Eq. (8) is virtually zero. The next step is to select parameter *k* in the growth quantifier. For this analysis data on stems and fruit are grouped together as the structural component (*Y_S+F_ = Y_S_+Y_F_*), which leads to
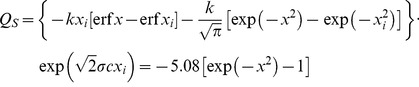
(9)for *k* = 9.0. Regression analysis of data in Table 1 leads to



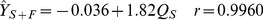
(10)Parameter *k* has been chosen to make the slopes in Eqs. (8) and (10) essentially equal. These correlations are shown in Figure 2, where the lines have been drawn from Eqs. (8) and (10). Growth curves in Figure 1 are drawn from Eqs. (8) and (10), where the curve for leaf fraction is drawn from *f_L_ = Q_L_/*(*Q_L_+Q_S_*).

The question naturally arises as to the procedure for simulation where only data for total biomass are available. In this particular case the total growth quantifier (*Q*) is given by 
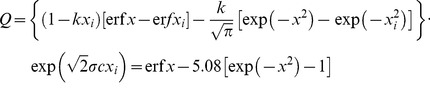
(11)


Linear regression of data in Table 1 leads to 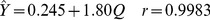
(12)which exhibits essentially the same slope as Eqs. (8) and (10). These results hinge on selection of parameter *k*.

**Figure 3:**
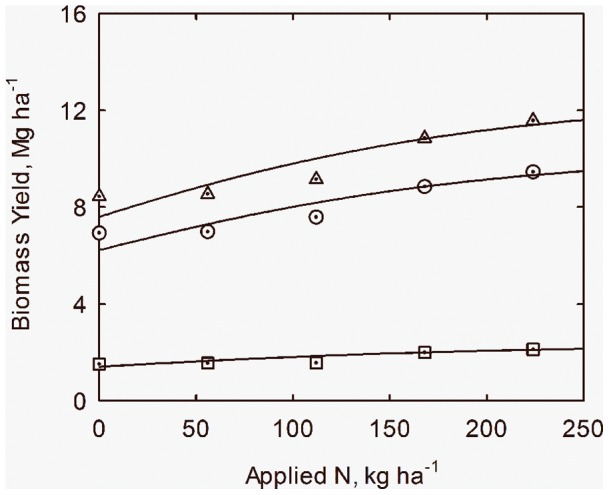
Response of biomass (*Y*) to applied nitrogen (*N*) for leaves and stems + fruit for cotton in San Joaquin Valley, CA. Data adapted from Fritschi et al. (2003). Curves drawn from Eqs. (16) through (18).

Response to applied N is listed in Table 2, model parameters are given in Table 3 and shown in Figure 3. The logistic model has been shown to describe this response for many crops [1], and can be written as

**Table 2:** Response of leaf (*Y_L_*), stem (*Y_S_*), and fruit (*Y_F_*) biomass to applied nitrogen (*N*) for cotton on Wasco sandy loam in San Joaquin Valley, CA (2000) [6].

*N*	*Y_L_*	*Y_S_*	*Y_F_*	*Y_S+F_*	*Z_y_*
kg ha^-1^	Mg ha^−1^	Mg ha^−1^	Mg ha^−1^	Mg ha^−1^	
0	1.51	1.72	5.21	6.93	0.751
56	1.55	1.74	5.25	6.99	0.778
112	1.56	1.96	5.63	7.59	1.07
168	1.99	2.48	6.37	8.85	1.88
224	2.12	2.49	6.97	9.46	2.55

**Table 3:** Logistic parameters for response of leaf (*Y_L_*), stem (*Y_S_*), and fruit (*Y_F_*) biomass to applied nitrogen (*N*) for cotton on Wasco sandy loam in San Joaquin Valley, CA (2000) [6].

Parameter	Value
*A_S+F_*, Mg ha^−1^	10.2
*B*	−0.44
*c_n_*, ha kg^−1^	0.00850
*R*	0.946



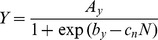
(13)in which *N* is applied nitrogen, kg ha^−1^; *Y* is biomass yield, Mg ha^−1^; *A_y_* is maximum yield at high N, Mg ha^−1^; *b_y_* is the intercept parameter for yield; and *c_n_* is the nitrogen response coefficient, ha kg^−1^. Note that the units on *c_n_* are the inverse of the units on *N*. This model can be linearized to the form
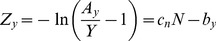
(14)For the case of cotton data focus is first on stems + fruit, where it is assumed that *A_S+F_* = 10.2 Mg ha^−1^ by visual inspection of Figure 3. Linear regression then leads to
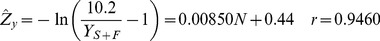
(15)which leads to the yield response equation



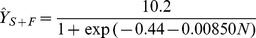
(16)The model for the leaf component is then written as
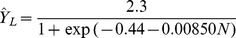
(17)where the same values of parameters *b_y_* and *c_n_* are assumed as for stems + fruit and with *A_y_* = 2.3 Mg ha^−1^ optimized according to regression theory. It follows that the model for total biomass (*Y*) becomes



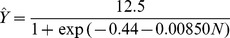
(18)The curves in Figure 3 are drawn from Eqs. (16) through (18). Equation (13) can be written in the alternate form
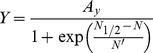
(19)in which *N*_1/2_ *= b_y_*/*c_n_* is applied N to achieve 50% of maximum yield, kg ha^−1^; and 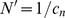 is defined as characteristic N for the system, kg ha^−1^. For the present case 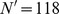 kg ha^−1^ and *N*_1/2_ = 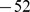 kg ha^−1^. The negative value indicates that more than enough soil N is already present to achieve 50% of maximum yield.

### Alabama Study

Data for this analysis are taken from a study in northern Alabama by Mullins and Burmester [7]. Four cultivars (‘Deltapine 90′, ‘Stoneville 825′, ‘Coker 315′, and ‘Paymaster 145′) were grown on Decatur silt loam (clayey, thermic, kaolinitic Rhodic Paleudult) in 1986 and 1987. The latter year is chosen for analysis where applied N was 78 kg ha^−1^. No supplemental irrigation was provided. All treatments were replicated four times. Planting date was April 20 (*t* = 15.9 wk). Plant samples were analyzed for dry matter as well as plant N, P, and K. Averages for the four cultivars are used in this analysis.

**Figure 4:**
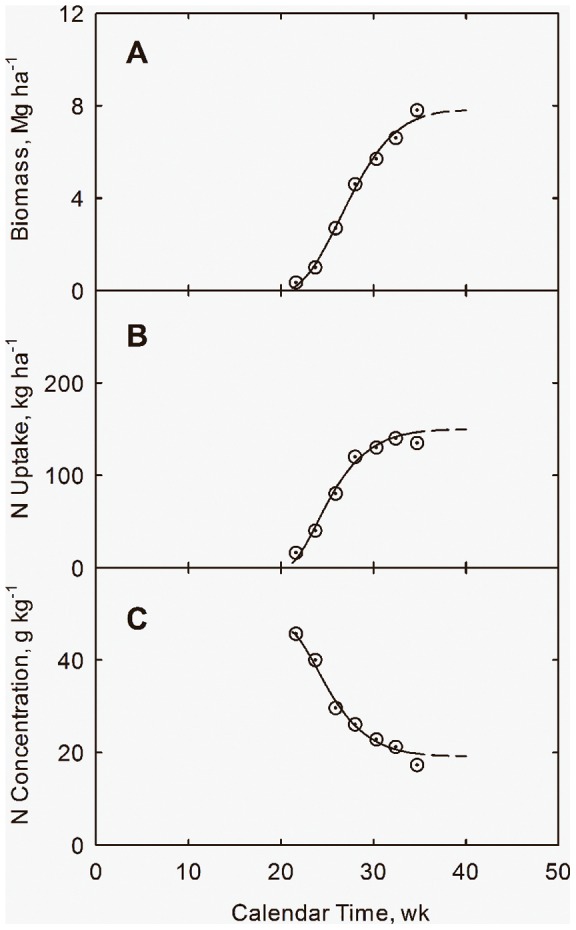
Growth response with calendar time (*t*) for cotton in northern Alabama. Accumulation of biomass (*Y*) (**A**), plant N uptake (*N_u_*) **B**), and plant N concentration (*N_c_*) (**C**) with calendar time. Data adapted from Mullins and Burmester (1990). Curves drawn from Eqs. (20) through (22), (25), and (28).

Results are listed in Table 4 and shown in Figure 4. Model parameters are assumed the same as for California and are: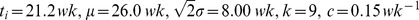 This leads to the equations
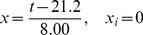
(20)


**Table 4:** Accumulation of biomass (*Y*), plant N (*N_u_*), plant P (*P_u_*), and plant K (*K_u_*) with calendar time (*t*) for cotton on Decatur silt loam in northern Alabama (1987) [7].

*t*	*x*	erf *x*	exp*(−x^2^)*	*Q*	*Y*	*N_u_*	*P_u_*	*K_u_*
wk					Mg ha^−1^	kg ha^−1^	kg ha^−1^	kg ha^−1^
21.2	0.000	0.000	1.0000	0.000	-	-	-	-
21.6	0.050	0.056	0.9975	0.069	0.35	16	1.5	9
23.7	0.312	0.341	0.907	0.813	1.0	40	3.4	20
25.9	0.588	0.595	0.708	2.08	2.7	80	7.7	60
28.0	0.850	0.771	0.486	3.38	4.6	120	7.4	90
30.3	1.138	0.892	0.274	4.58	5.7	130	15	100
32.4	1.400	0.952	0.141	5.32	6.6	140	15	90
34.7	1.688	0.9830	0.0580	5.77	7.8	135	16	110
	∞	1	0	6.08	-	-	-	-



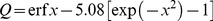
(21)Linear regression of data in Table 4 leads to
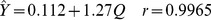
(22)


**Figure 5:**
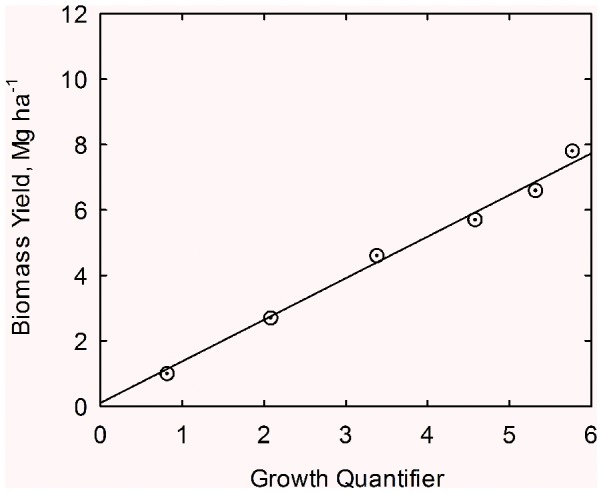
Correlation of biomass (*Y*) with the growth quantifier (*Q*) for cotton in northern Alabama. Yield data adapted from Mullins and Burmester (1990). Line drawn from Eq. (22).

Note similarity of the slope with that from California at low N (1.27 vs. 1.33 Mg ha^−1^). The line in Figure 5 is drawn from Eq. (22).

**Figure 6:**
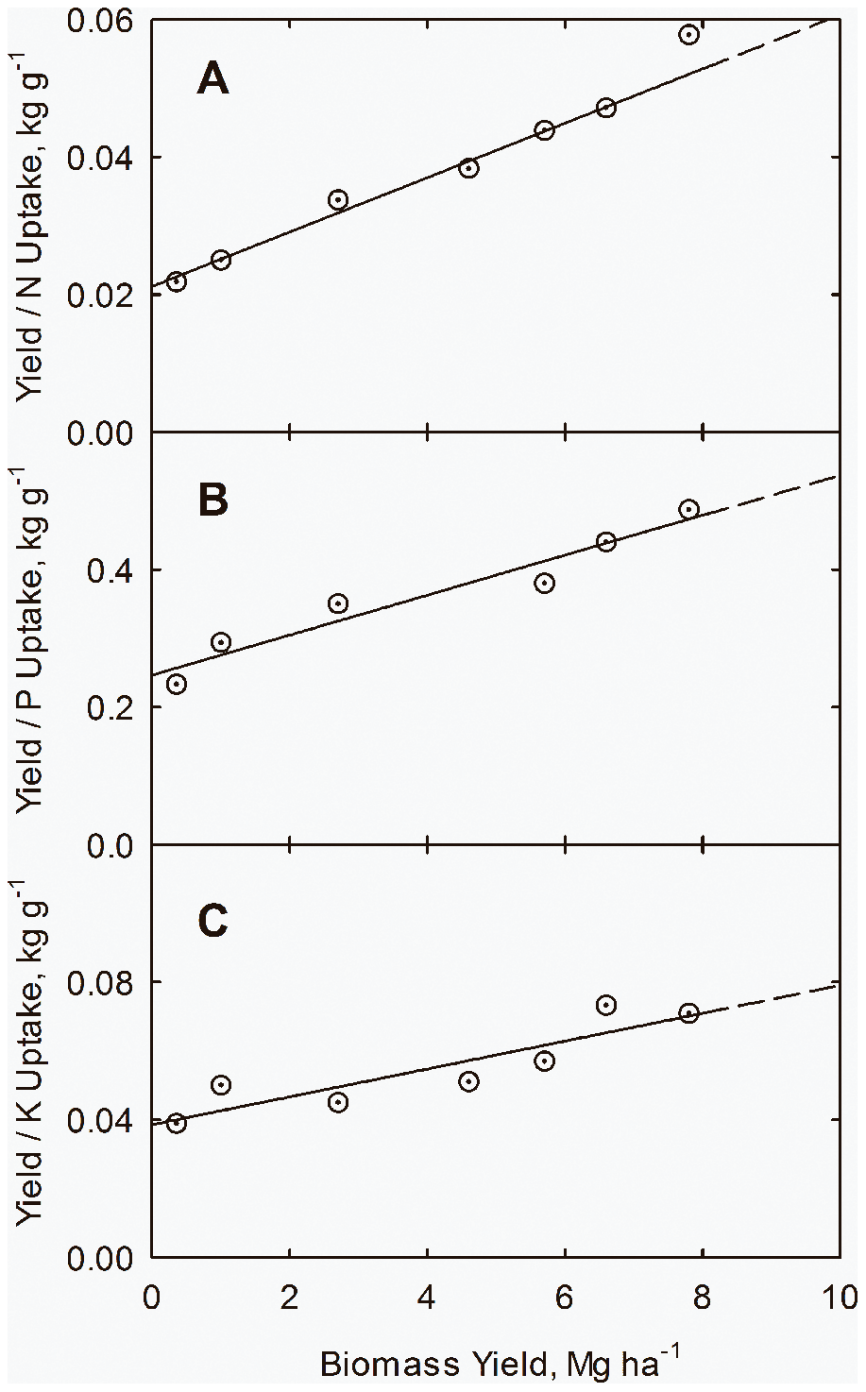
Correlation of nutrient uptake ratios with yield (*Y*) for cotton in northern Alabama. Correlation of N (**A**), P (**B**) and K (**C**) nutrient uptake ratios (*Y/N_u_, Y/P_u_, Y/K_u_*) with yield. Data from Mullins and Burmester (1990). Lines drawn from Eqs. (25) through (27).

**Figure 7:**
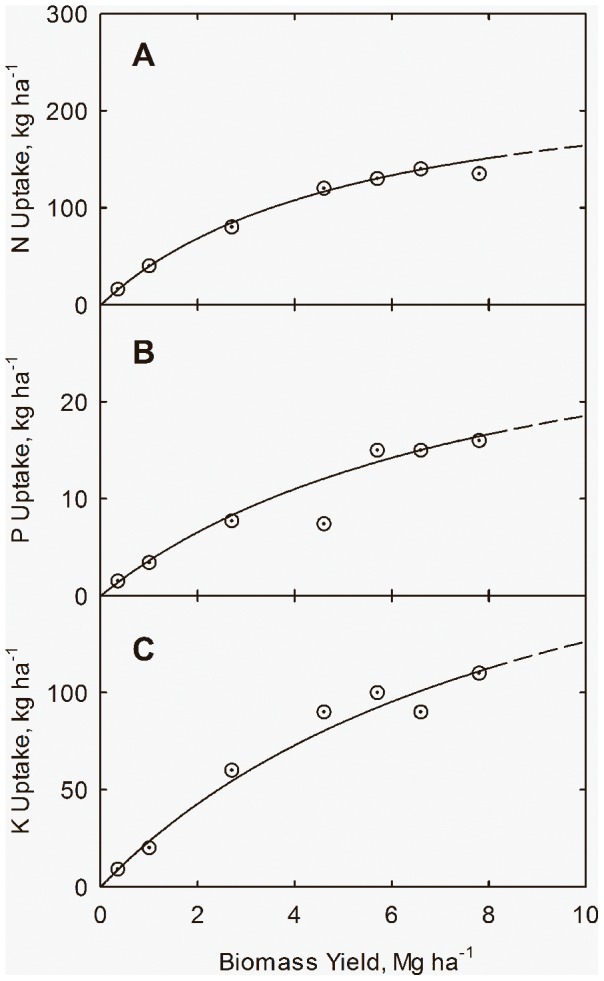
Coupling of plant nutrient uptake with biomass yield (*Y*) for cotton in northern Alabama. N uptake (*N_u_*) (**A**), plant P uptake (*P_u_*) (**B**), and plant K uptake (*K_u_*) (**C**) with yield. Data adapted from Mullins and Burmester (1990). Curves drawn from Eqs. (25) through (27).

Plant nutrient uptake can be related to biomass through the hyperbolic phase relation
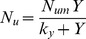
(23)in which *N_u_* is accumulated plant nutrient (N, P, or K), kg ha^−1^; *N_um_* is potential maximum plant nutrient, kg ha^−1^; and *k_y_* is the yield response coefficient, Mg ha^−1^. Equation (23) can easily be rearranged to the linear form



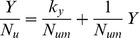
(24)Linear regression of data in Table 3 leads to the equations 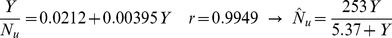
(25)

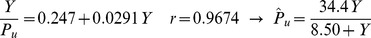
(26)

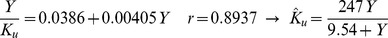
(27)for plant uptake of nitrogen (*N_u_*), phosphorus (*P_u_*), and potassium (*K_u_*), respectively. The lines and curves in Figures 6 and 7 are drawn from Eqs. (25) through (27). The curves in Figure 7 are drawn from Eqs. (25) through (27), and plant N concentration (*N_c_*) from 
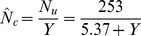
(28)


## Discussion

There is good agreement, in form and description, between the expanded growth model and field data from California and Alabama. Furthermore, all model parameters were common for the two sites with the exception of the yield factor *A* which accounts for differences in soil types, environmental conditions, fertilizer levels, and plant populations. Since biomass and plant nutrients are accumulating with calendar time the system involves rate processes. We conclude that accumulation of biomass with time by photosynthesis is the rate limiting process, and that coupling of plant nutrients and biomass proceeds in virtual equilibrium as assumed in the phase relations. This conclusion is borne out by the memoir by Overman and Scholtz [2] which provides a detailed derivation of the expanded growth model employed in the present analysis.

Since biomass and plant nutrients are accumulating with calendar time the system involves rate processes. We conclude that accumulation of biomass with time by photosynthesis is the rate limiting process, and that coupling of plant nutrients and biomass proceeds in virtual equilibrium as assumed in the phase relations. This conclusion is borne out by the memoir by Overman and Scholtz [2] which provides a detailed derivation of the expanded growth model employed in the present analysis.

An excellent discussion of photosynthesis has been presented by Morton [8], with emphasis on what has been learned and what remains as open questions.

All of our work on crop growth and yield has been founded on five organizing principles (codified in the Preface) [1]: (1) patterns, (2) relations, (3) connections, (4) consistency, and (5) beauty. *Patterns* refer to arithmetic and geometric forms. It is common practice to show results in graphical format, with the response variable on the vertical axis and the control variable on the horizontal axis. This allows examination of possible trends (linear, exponential, logistic, etc.) and of scatter in the data. Examples include response of biomass yield to applied nutrient (such as nitrogen) and response of biomass yield to calendar time. *Relations* refer to mathematical models such as exponential, logistic, hyperbolic, probability, etc. A model can be either derived from basic theory or based on intuition. Both have been used successfully in science. *Connections* refer to coupling between or among components of a system. Examples include biomass and nutrient accumulation by plants. In physics it can include such variables as pressure and volume of a gas, position and momentum of a mechanical system, as well as electrical and magnetic potential. It often takes the form of a phase relation between two response variables. *Consistency* refers to either internal consistency or among different sets of data for studies of the same variables under different environmental or experimental conditions. An example might include response of a particular crop species to applied nitrogen for different years at the same location or from different locations with different soil types. *Beauty* refers to mathematical beauty. Examples include the law of falling bodies and inertia by Galileo, the three laws of planetary motion of Kepler, the laws of motion and gravitation of Newton which unified terrestrial and celestial motion from Galileo and Kepler, the laws of electromagnetism formulated by Maxwell which unified the theories of Faraday and Coulomb. The ultimate achievement of mathematical beauty occurred through a theorem proven in 1915 by the mathematician Emmy Noether which establishes the connection between mathematical symmetry and a conservation principle for a dynamic system [9]. All of these principles have been used in our work in some form.
